# Low night temperature at veraison enhances the accumulation of anthocyanins in Corvina grapes (*Vitis Vinifera* L.)

**DOI:** 10.1038/s41598-018-26921-4

**Published:** 2018-06-07

**Authors:** Federica Gaiotti, Chiara Pastore, Ilaria Filippetti, Lorenzo Lovat, Nicola Belfiore, Diego Tomasi

**Affiliations:** 1 CREA - Council for Agricultural Research and Economics, Viticulture Research Centre, Via 28 Aprile, 26, 31015 Conegliano, Italy; 2 Department of Agricultural Sciences, University of Bologna, Viale Fanin, 46, 40127 Bologna, Italy

## Abstract

Climate change is a major concern in grape production worldwide. Nights have been warming much faster than the days, raising attention on the effect of night temperatures on grape and wine composition. In this study we evaluated the effect of night temperatures on grape coloration in the cv. Corvina (*Vitis vinifera* L.). In 2015 and 2016 potted plants were cooled overnight (10–11 °C) during two berry ripening phases, veraison (TV) or post-veraison (TPV), and compared to control vines (C) grown at ambient night temperature (15–20 °C on average). Cooling treatment around veraison (TV) hastened berry anthocyanin accumulation, while the same treatment applied after veraison (TPV) was ineffective. Molecular analysis revealed an increased transcription of four key genes in anthocyanin biosynthesis (CHS3, F3H1, MYBA1 and UFGT) in TV treatment. These results suggest that the anthocyanin biosynthesis capacity was enhanced by cool nights during veraison. However, since the gene expression was not always temporally correlated to the increase in anthocyanin concentration, we speculate on the presence of mechanisms, such as enzymatic regulation or anthocyanin transport, which may contribute in determining the anthocyanin accumulation under low night temperatures.

## Introduction

There is mounting evidence that the current changes in climate across the Northern Hemisphere will continue in the future and affect temperature, precipitation, and atmospheric CO_2_ concentration. The global temperature is forecast to increase continuously 1–3.7 °C by the end of the 21^st^ century^1^. Night temperature has increased faster than day temperature at global scales^2^ and between 1950 and 1993, minimum temperatures have increased at about twice the rate of maximum temperatures^1^. Therefore, in the future plants will be exposed to warmer nights, which could greatly influence crop yield and vegetation dynamics as well as ecosystem biodiversity, structure and productivity^3^.

In recent years, several researches have focused on the effects of night temperatures on the growth and/or yield of annual plants such as wheat^4^, cotton^5^, soybean^6^, sorghum^7^ and rice^8–10^, where it is reported that the increase in minimum temperatures adversely affects yield and quality more than high daytime temperature^11^. On fruits, the effect of night temperature is mainly linked to the maturation rate and quality and many studies have been conducted on apple and pear^12,13^, cherry^14^, strawberry^15,16^ and grape^17–24^.

Grapevine is the most widely grown fruit crop in the world, covering approximately 7.6 million hectares in 2016, producing more than 267 million hectolitres of wine (http://www.oiv.int) and it is cultivated on six out of seven continents, between latitudes 4° and 51° in the Northern Hemisphere and between latitudes 6° and 45° in the Southern Hemisphere^25^. The global range of grape growing climate zones is about 10 °C (between 12 °C and 22 °C growing season average temperatures), and even narrower for premium wine quality grapes^26^. Projections suggest that rising global temperatures will lead to a significant redistribution of the grape growing regions throughout the world^26,27^. Impacts will be negative for the areas that are already teetering on being too hot for quality grape production, while areas that were historically too cold are reaching or are projected to reach in the near future climate conditions suitable for viticulture^26,27^.

The minimum temperature for grapevine physiological activity is commonly estimated to be 10 °C and, depending on genotype, its variation before berry development can affect the net photosynthetic rate^19,23^ and impact flower development and fruit set^24,28^. During berry ripening, night temperature usually has relatively little effect on total soluble solids^17^, but can strongly affect acidity, aroma and anthocyanins^29,30^. It was reported that night temperatures below 15 °C increase acidity and that between 5 °C and 15 °C enhance aroma concentration^29^. In the first research on the effect of night temperature on anthocyanin accumulation, Cardinal, Pinot noir and Tokay vines placed at cool night (15 °C) temperatures from beginning of veraison until harvest had much more intense coloration than fruits ripened at warm nights (25 °C) and it was reported that the influence of night temperature on fruit coloration was dependent, at least in part, on the day temperature and on the differential between day and night temperatures^17^. More recently, Mori *et al*.^20^ showed that anthocyanin accumulation in the skin of Darkridge (*Vitis vinifera* L. x *Vitis labrusca* L.) berries placed prior to veraison (beginning of berry softening) at high night temperatures (30 °C continuous) was lower than that of berries grown at low night temperatures (30 °C daytime/15 °C night-time). This effect could be linked to the inhibition of gene expression of several genes involved in anthocyanin biosynthesis and to the decrease in PAL and UFGT activities detected in berries grown at 30 °C. Also, in Pinot Noir anthocyanin accumulation in berry skins grown under high night temperatures was lower than in berries grown under low night temperatures around 15 °C^21^. Cohen *et al*.^31^ investigated the accumulation of phenolics in grape berries cv. Merlot in response to various temperature treatments. Berries heated at night (+8 °C in comparison to ambient temperature, in which minimum temperature ranged from 2.5 to 6.4 °C) showed in this case higher anthocyanin concentration at harvest, compared to berries ripened at ambient temperature. In accordance with long-term temperatures studies, the application of a short stress period of two hours at 37 °C imposed after sunset on one-year-old microvine plants at beginning of veraison, reduced total anthocyanin content by a factor of 2.5 in comparison with untreated vines, ripened at a night temperature of 12 °C^32^.

Corvina is an indigenous cultivar of the Northern Italy, and it is used to produce wines such as Bardolino and Valpolicella, and fine wines such as Recioto and Amarone after a post-harvest withering process^33^. Corvina is considered a low coloured grape when compared to the average concentrations observed in the phenotyping screening performed on several European grapevine cultivars^34^. The Corvina historical production area covers the whole hillsides of the province of Verona, in the Veneto Region, extending from 50 m to 500 m above sea level. In the last half century this area has been subjected to a significant warming trend, with vine growing season average temperatures increasing 2.3 °C from 1964 to 2009^35^. Currently, maximum temperatures in the summer often reach 30–35 °C, while minimum temperatures frequently exceed 20 °C in the warmest months (http://www.arpa.veneto.it). Such high temperatures, that nowadays are a common occurrence in several vine growing areas worldwide, are on the limit for optimal ripening processes, in particular for anthocyanin biosynthesis, which has been reported to be favored under mild day temperatures (25 °C)^17,36,37^, and under night temperatures below 15 °C^20,21^.

A strategy proposed to cope with the effect of global warming, is to consider shifting vine cultivation to cooler areas, moving poleward or at higher elevation^27,38^. In these new conditions, benefits for grape quality achievable by more moderate day temperatures are well recognized. However, how grape ripening and coloration are affected by cool night temperatures is still uncertain.

On these bases, considering that to date most recent researches have focused essentially on assessing the effects of daily temperatures on berry composition, and in particular on the accumulation of anthocyanins^39–42^, we set up a study to evaluate through an integrated biochemical and molecular approach the effect of low night temperatures (10-11 °C) on anthocyanin accumulation in the cv. Corvina in two different ripening phases: during veraison or from the end of veraison until full ripening.

## Results

### Climatic conditions of 2015 and 2016 growing seasons

**Figure 1:**
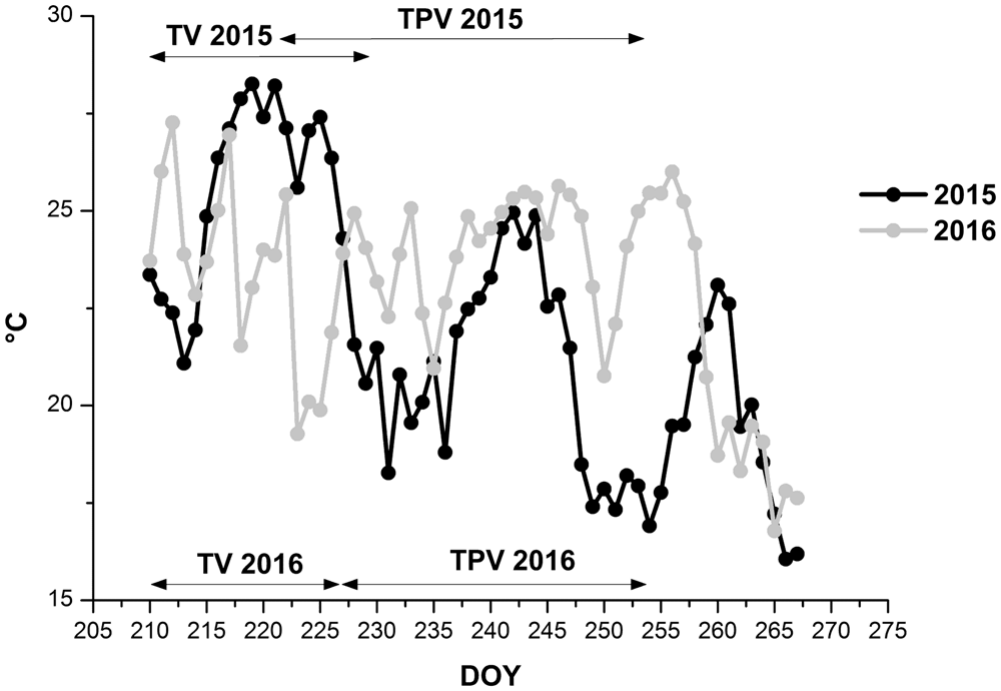
Trends in mean air temperature from the beginning of the experiment (pre-veraison) to harvest in 2015 and 2016 seasons. The duration of the low night temperature treatments during veraison (TV) or post veraison (TPV) periods is indicated by black double arrows.

The 2015 and 2016 growing seasons were very different from one another and the air thermal summation, expressed as degree-days (GDD, °C) above base 10 °C from DOY 210 to 267, mainly corresponding to the ripening period for both years, resulted as 669 °C in 2015 and 722 °C in 2016, indicating the latter as the warmer year of the trial. The two seasons also strongly differed in the trends of mean (Fig. 1), minimum and maximum temperature (Supplementary Fig. S1). In 2015 the warmest period was during veraison, between 218–226 DOY, due to maximum temperatures always exceeding 30 °C with peaks around 35 °C (Supplementary Fig. S1). In 2016, an opposite trend was recorded, with the average temperature being lower than the previous year during veraison (214–228 DOY), reaching values below 20 °C, and higher after veraison, in particular from 228 to 258 DOY (Fig. 1), despite no maximum temperatures above 35 °C (Supplementary Fig. S1). In both years minimum temperatures ranged between 15 and 20 °C for almost all ripening period, except during veraison in 2015, when they frequently exceeded 20 °C (Supplementary Fig. S1).

### Effects of treatments and season on berry temperature

**Figure 2:**
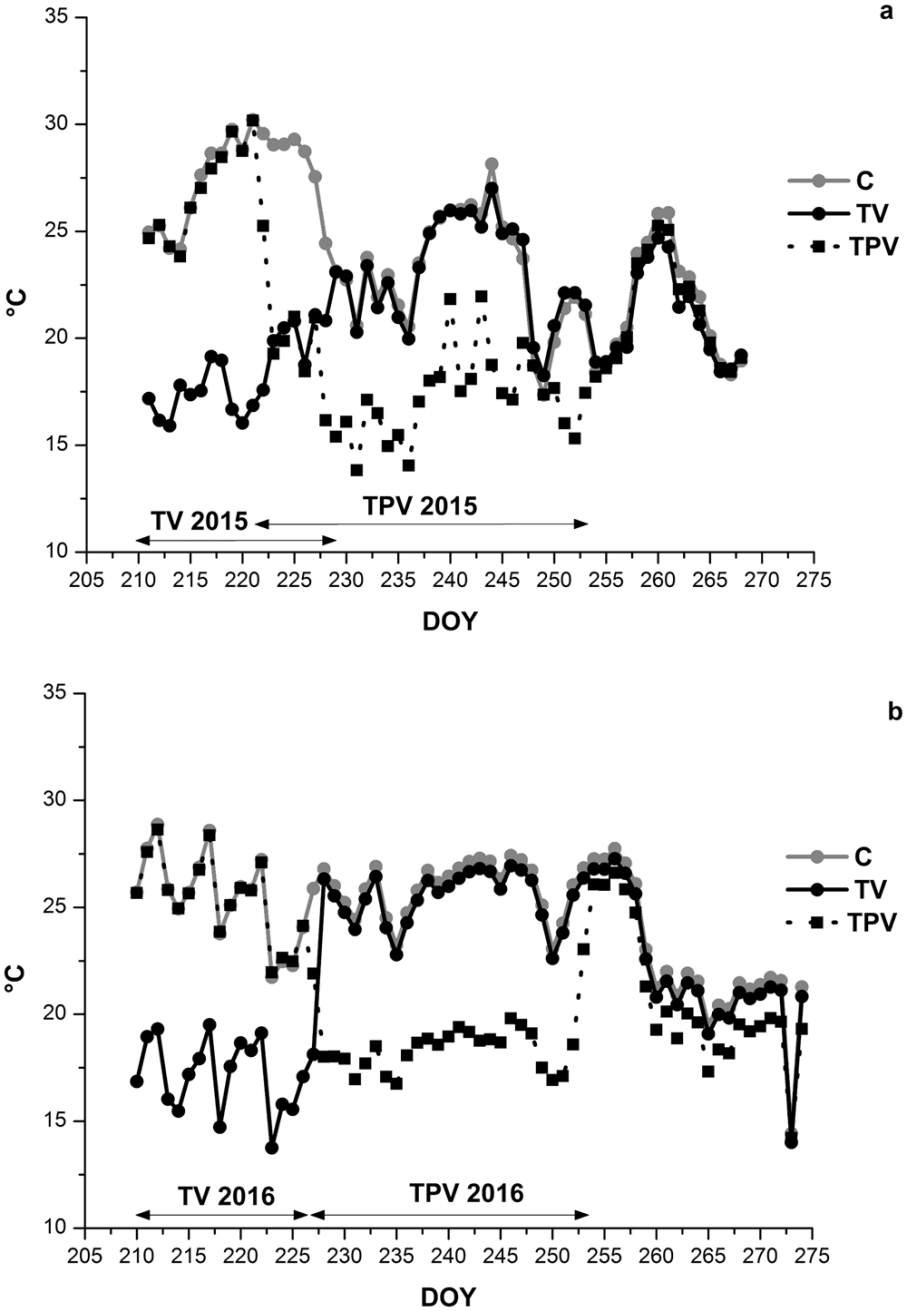
Trends in mean berry temperature for Corvina vines treated at low temperature in the night (TV and TPV) and for the control (C), from the beginning of the experiment (pre-veraison) to harvest in 2015 (**a**) and 2016 (**b**) seasons. The duration of the low night temperature treatments during veraison (TV) or post veraison (TPV) periods is indicated by black double arrows.

During the imposed treatments, in both years TV and TPV treated berries reached a minimum temperature of around 10 °C in the climatic chamber during the night, while C berries minimum berry temperature was between 15 and 25 °C (Supplementary Fig. S2). Maximum temperatures were not affected by the treatments and displayed similar trends among C, TV and TPV berries showing values from 20 to 40 °C in 2015 and from 20 to 35 °C in 2016 (Supplementary Fig. S2) with peaks around veraison in 2015 when berries were subjected to maximum temperatures from 35 to 40 °C. Maximum berry temperature was almost constant in 2016 ranging mainly from 30° and 35 °C, with lower or higher values being detected only a few times. In both years there was a decrease in the minimum and maximum berry temperatures close to harvest time (Supplementary Fig. S2). As expected, mean berry temperatures were strongly affected by the treatments and showed a trend that exactly mirrored the one observed for minimum temperatures in both years, with TV and TPV having similar values to C when outside the climatic chamber. In 2015 and 2016 mean berry temperatures never exceeded 30 °C but, as expected from 2015 air temperature, mean berry temperature was higher around veraison in this year, while in 2016 it was more stable throughout ripening (Fig. 2a,b).

The differences induced by the treatments in minimum berry temperatures strongly affected the thermal gap between daytime and night-time temperature (T_max_ – T_min_) in TV and TPV treatments in comparison to C, in which T_max_ – T_min_ was around 11 °C throughout ripening in both years. During the period of application of low night temperature, T_max_ – T_min_ values in TV were 25 °C and 21 °C in 2015 and 2016 respectively, while in TPV similar values, around 21 °C, were detected in both years.

### Vine physiological response and berry technological maturity

Vine physiological parameters were measured on two dates (in pre-veraison, before the beginning of the night-temperature treatment, and at mid maturation stage), with the double purpose of assessing whether seasonal or imposed night thermal regimes differently affect plant water status and photosynthetic activity of the vines.

Comparing the treatments, no differences were found for stem water potential, nor for the assimilation rate on any date (Table 1). Data recorded at mid maturation stage (DOY 244 in 2015, DOY 249 in 2016) showed similar values for TV, TPV and the control, indicating that no photosynthetic performance variation occurred in plants exposed to cool nights.

**Table 1:** Values of midday stem water potential (Ψ_stem_) and leaf net assimilation rate (An) for the treatments and the control, recorded in pre-veraison, before the beginning of the experiment, and at mid maturation stage, in the two seasons of study (2015–2016).

	DOY	Ψ_stem_(Mpa)				An (μmol CO_2_ m^2^ s^−1^)			
		C	TV	TPV	Sign	C	TV	TPV	Sign
2015	198	−1,1	−1,1	−1,1	ns	7,2	7,1	7,2	ns
	244	−0,9	−0,9	−1,0	ns	9,4	8,6	9,0	ns
2016	204	−0,9	−0,9	−0,8	ns	12,4	12,0	12,4	ns
	249	−1,0	−1,0	−1,1	ns	9,5	10,5	10,7	ns

Data are means for measurements on six fully exposed leaves per treatment. Treatment effect is reported (ns: not significant).

Comparing the two studied seasons, year showed a significant effect on both physiological parameters (p < 0.05). Indeed, the pre-veraison phase was notably hotter in 2015 than 2016, affecting the stem water potential and photosynthetic rates, which showed lower values for all treatments compared to those recorded at the same stage in 2016.

Berry ripening was affected both by seasonal and imposed thermal ranges. Air temperature, and consequently mean berry temperature, around veraison were higher in 2015 than 2016. In the former season, application of low night temperature during the early stage of ripening delayed veraison, with TV completing this phase 6 days after TPV and C. For this reason, TV and TPV treatments partially overlap in 2015. This did not happen in 2016, when ripening proceeded in a similar way in the three treatments.

**Figure 3:**
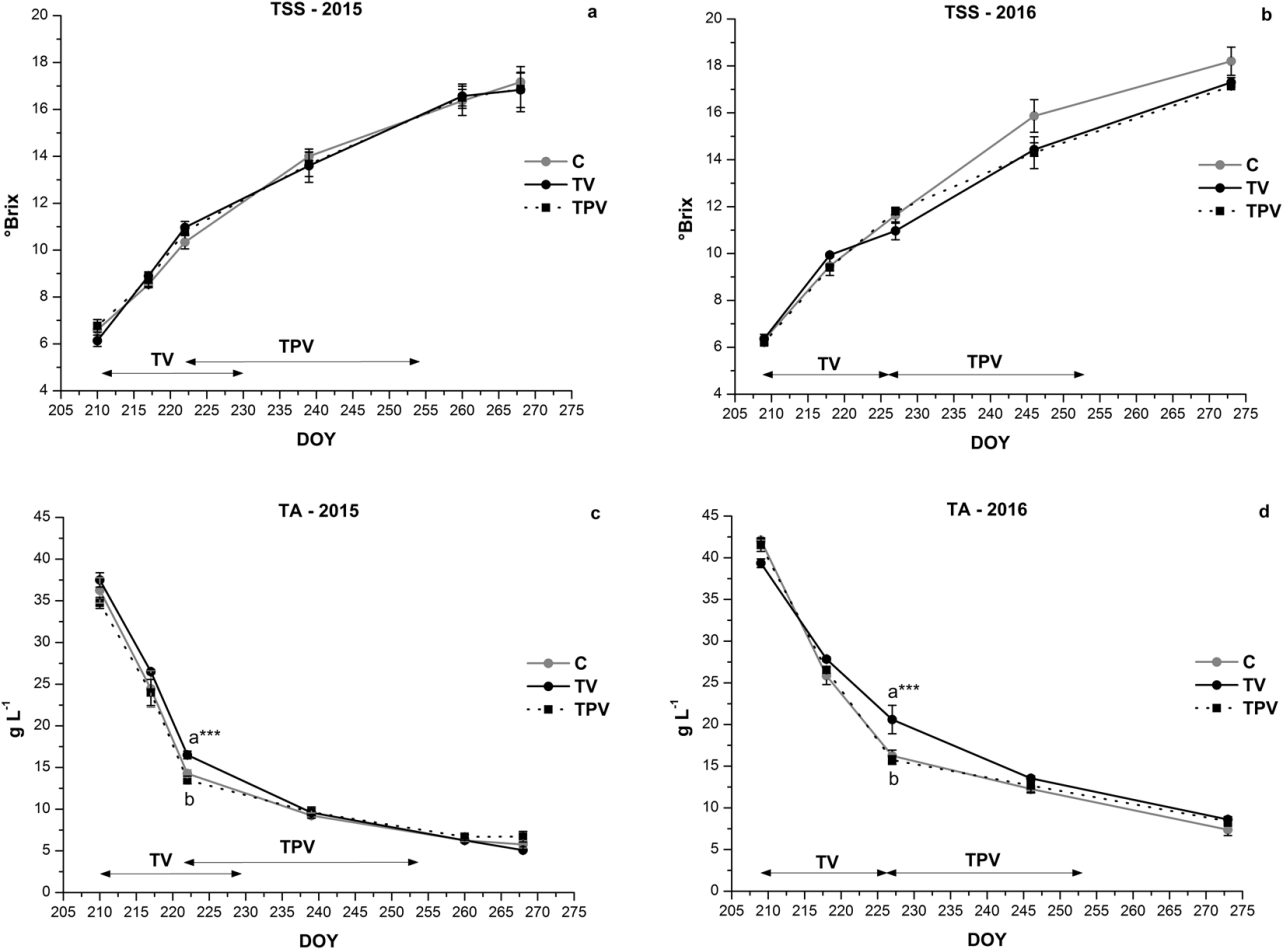
Evolution of total soluble solid content (TSS; **a**,**b**) and titratable acidity (TA; **c**,**d**) for Corvina vines treated at low temperature in the night and for the control during the ripening, in 2015 and 2016. Error bars indicate the mean SE (*n* = 3). Means followed by different letters differ significantly, as calculated by Tukey statistical analysis (*, **, ***p ≤ 0.05, 0.01, 0.001, respectively). The duration of the low night temperature periods is indicated by black double arrows, for TV and TPV treatments.

In both seasons, there were no significant differences between treated vines and the control for the evolution of berry weight (data not shown), nor for total soluble solids (TSS) accumulation during the experimental period (Fig. 3a,b). Grapes from TV and TPV ripened at a near linear rate, similarly to C, continuing unabated during the periods of low night temperature exposure. The sugar ripening rate for all treatments averaged 0.17° Brix day^−1^ in both seasons (r^2^ = 0,95 in 2015, r^2^ = 0.93 in 2016, P < 0.05), with slightly higher values for C in 2016 (0.18° Brix day^−1^, r^2^ = 0.92, P < 0.05). Total soluble solids content at harvest was similar across treatments and years, averaging 16.9° Brix in 2015 and 17.5° Brix in 2016.

In contrast to grape sugar content, some significant differences were observed for acid concentration trends over the two seasons (Fig. 3c,d). Titratable acidity (TA) declined linearly for all treatments during ripening, but exposure to low night temperature before and during veraison appeared to slow down acid degradation. During this period, titratable acidity in TV displayed an average decrease of 1.2 g  L^−1^ day^−1^ (r^2^ = 0.83, P < 0.05) while in TPV and C it declined at a rate of 1.5 g  L^−1^ day^−1^ (r^2^ = 0.83, P < 0.05). In both seasons, TV showed significantly higher acid concentrations at the end of veraison than TPV and C. No effect was observed on titratable acidity when low night temperature was applied during the second ripening stage, after the end of veraison. The difference observed at veraison levelled off through mid-late ripening stage, and acid concentration at harvest displayed similar values for treated vines and control in both seasons, averaging 5.9 g L^−1^ in 2015 and 8.1 g L^−1^ in 2016.

### Anthocyanin accumulation and profile

**Figure 4:**
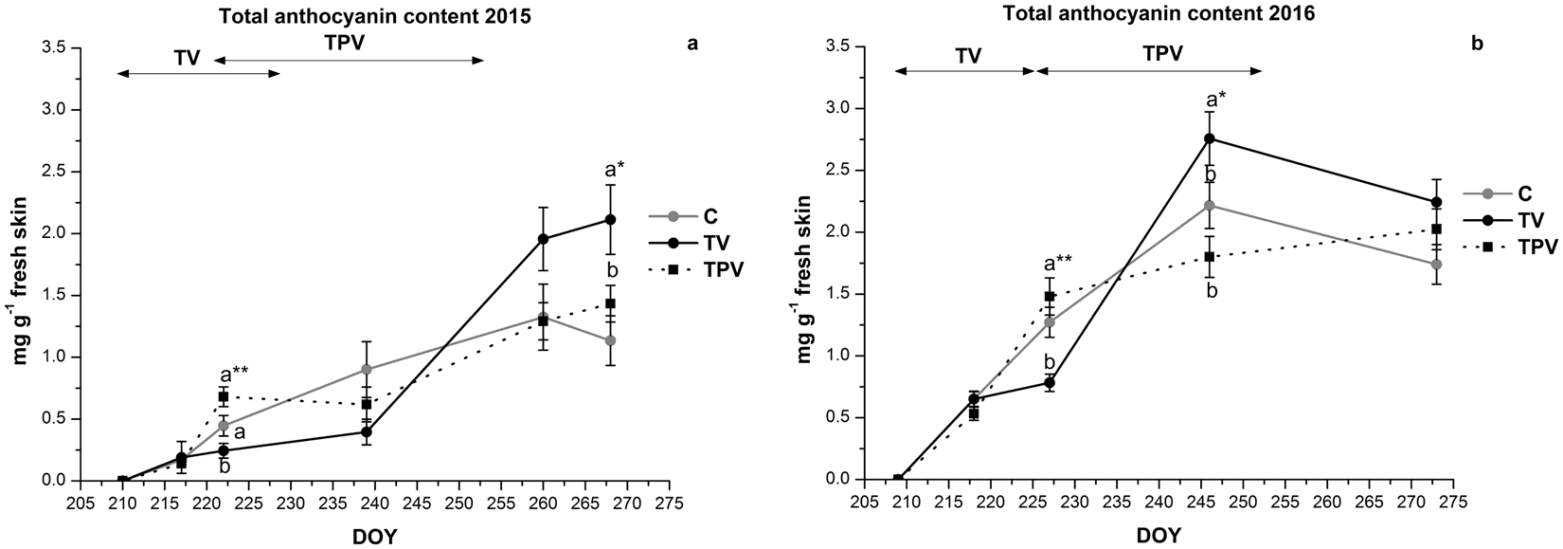
Trends of total anthocyanin concentrations in the skin of Corvina grapes treated under low night temperature (TV, TPV) and in the control (C), during the experimental period in 2015 (**a**) and 2016 (**b**). Error bars indicate the mean SE (*n* = 3). Means followed by different letters differ significantly, as calculated by Tukey statistical analysis (*, **p ≤ 0.05, 0.01, respectively). The duration of the low night temperature periods is indicated by black double arrows, for TP and TPV treatments.

Total anthocyanin concentration and profiles of the single anthocyanins were determined on samples collected during ripening in 2015 and 2016 (Fig. 4a,b and Supplementary Fig. S3).

The total anthocyanin content in skins of Corvina berries increased during ripening on all vines, showing significant differences due to temperature treatments (Fig. 4a,b). Low night-time temperature applied in the early ripening phase (TV treatment) significantly reduced the anthocyanin accumulation in both seasons, and at the end of veraison, the anthocyanin concentration in TV berries was significantly lower than that of TPV and C. Surprisingly, when TV vines were re-exposed to external night air temperature (DOY 228 in 2015, DOY 227 in 2016), anthocyanin concentration started increasing sharply, reaching values higher than TPV and C at harvest (Fig. 4a,b). Although differences were more evident in 2015, when higher air temperature during veraison exerted a greater impact on berry ripening, a similar trend with increased anthocyanin accumulation rates following the exposure to TV treatment was also observed in 2016.

The application of low night-time temperature after the end of veraison did not show any significant effect on colour compounds, with TPV displaying anthocyanin concentrations similar to that of the control at all sampling dates.

All single anthocyanins, either mono or di-glucosides, and their total acylated fraction showed accumulation trends similar to those observed for the total anthocyanin pool during ripening (Supplementary Fig. S3). Only a small effect of temperature treatments was observed on the anthocyanin profile at harvest (Table 2). Malvidin 3-glucoside and peonidin 3-glucoside were the most abundant anthocyanins, accounting for 65% on average of total skin anthocyanins in all treatments and in both seasons. Delphinidin 3-glucoside and petunidin 3-glucoside showed a slightly higher percentage in TV and TPV than C, although differences were significant only in 2016, while in 2015 an increase in acylated anthocyanins was detected in C, in comparison to TV and TPV.

**Table 2:** Mean values of individual and total acylated anthocyanins, in Corvina berry skins at harvest in 2015 and 2016, for treated vines and the control.

Year	Treatment	Anthocyanin profile (% on total anthocyanin content)								
		Del-3-G		Cya-3-G	Peo-3-G	Pet-3-G		Mal-3-G	Tot acylated anthocyanins	
2015	C	1,5		4,6	20,6	2,8		39,3	31,3	a
	TV	2,2		7,4	32,2	3,4		34,7	20,1	b
	TPV	2,4		4,3	23,3	3,9		43,2	22,9	b
*Sig*		*ns*		*ns*	*ns*	*ns*		*ns*	*	
2016	C	1,3	b	5,5	35,8	2,8	b	30,5	24,0	
	TV	2,4	a	5,6	27,9	4,2	a	36,0	23,9	
	TPV	2,4	a	5,8	33,2	3,8	a	34,7	20,1	
Sig		*		ns	ns	*		ns	ns	

Data are expressed as % on the total anthocyanin content. Means followed by different letters differ significantly as attested by Tukey test (*p ≤ 0.05; ns = not significant).

### The application of low night temperature interacts with the season to differently affect genes involved in flavonoid and anthocyanin biosynthesis

**Figure 5:**
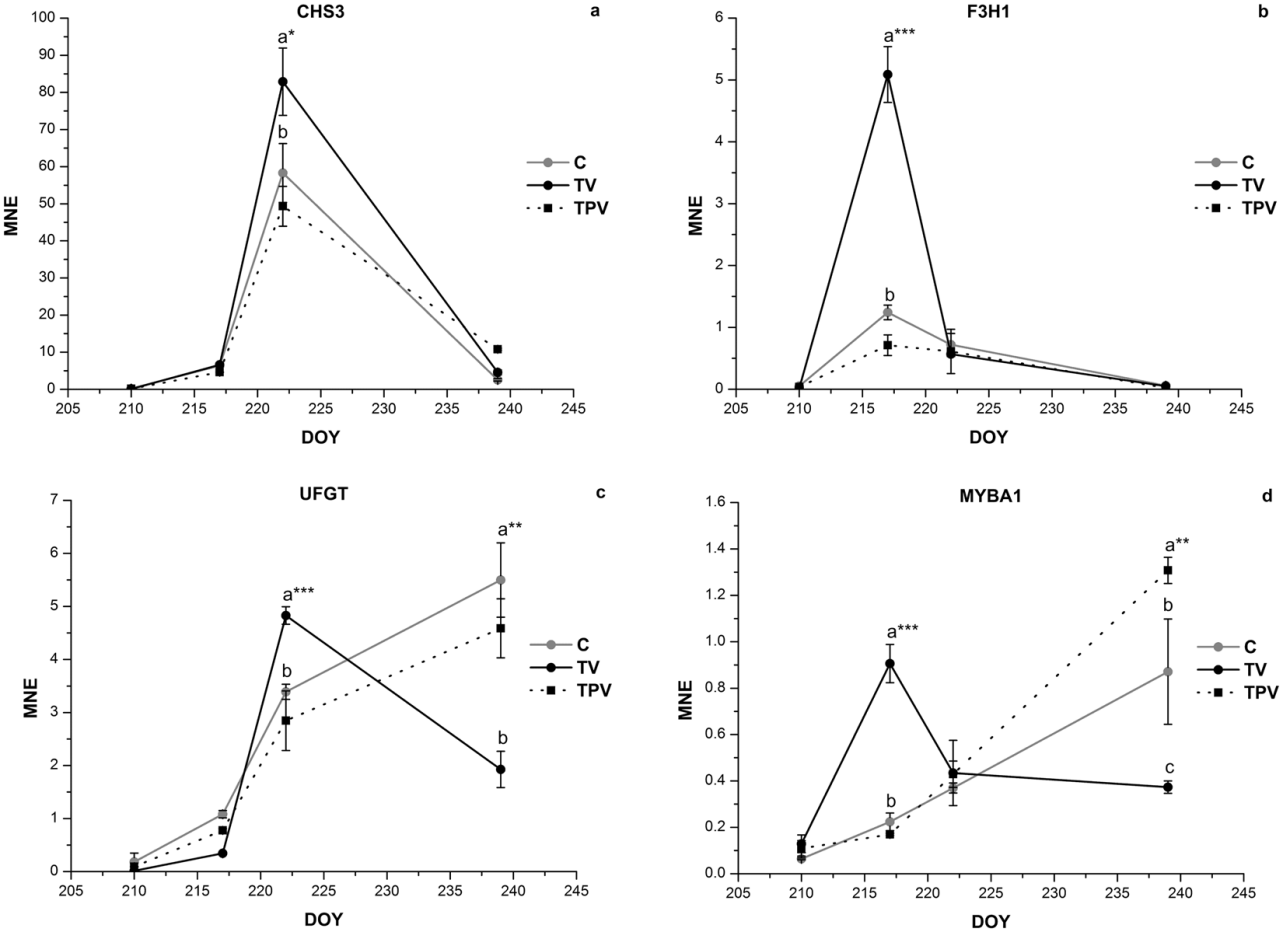
Expression profiles of CHS3 (**a**), F3H1 (**b**), UFGT (**c**) and MYBA1 (**d**) genes in the skin of Corvina grapes treated under low night temperature (TV, TPV) and in the control (C), during the experimental period in 2015. Real time RT-PCR data are reported as mean normalized expression (MNE) values, obtained using Ubiquitin-1 as reference gene. Error bars indicate the mean SE (*n* = 3). Means followed by different letters differ significantly, as calculated by Tukey test (*, **, ***p ≤ 0.05, 0.01, 0.001 respectively).

**Figure 6:**
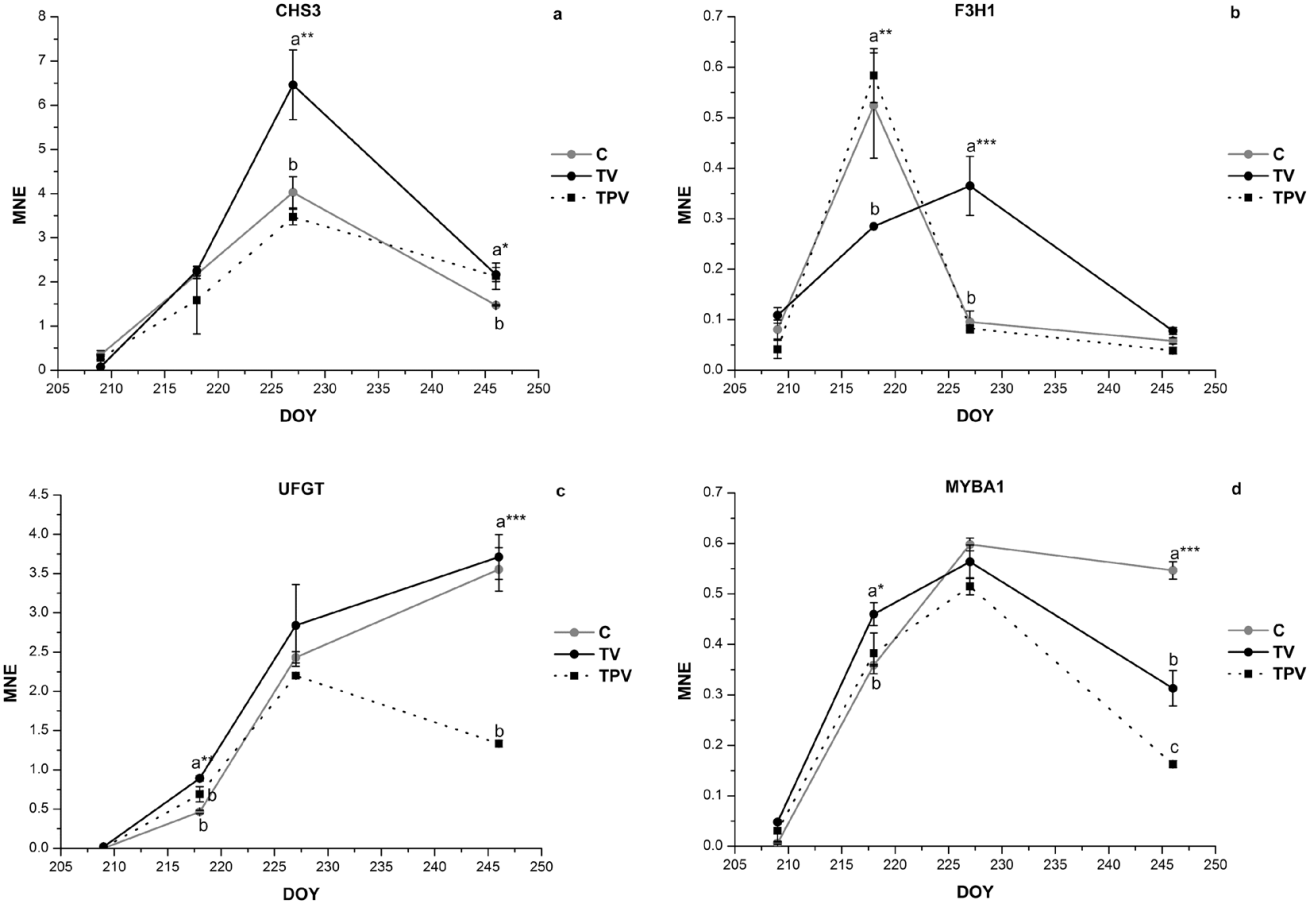
Expression profiles of CHS3 (**a**), F3H1 (**b**), UFGT (**c**) and MYBA1 (**d**) genes in the skin of Corvina grapes treated under low night temperature (TV, TPV) and in the control (C), during the experimental period in 2016. Real time RT-PCR data are reported as mean normalized expression (MNE) values, obtained using Ubiquitin-1 as reference gene. Error bars indicate the mean SE (*n* = 3). Means followed by different letters differ significantly, as calculated by Tukey test (*, **, ***p ≤ 0.05, 0.01, 0.001 respectively).

Gene expression studies on the main genes involved in flavonoid and anthocyanin biosynthesis in cv. Corvina berry skin under TV and TPV treatments were conducted on samples collected during ripening in 2015 and 2016 to elucidate the molecular mechanisms underlying the effect induced by low night temperature on anthocyanin accumulation (Figs 5, 6 and Supplementary Figs S4 and S5). Although with different trends during the two seasons, four genes showed an increase in expression in TV in comparison to C, regardless of the year: CHS3, an isoform of chalcone synthase, which catalyses the first step for the biosynthesis of both flavonoids and anthocyanins; F3H1, an isoform of flavanone 3-hydroxylase, which converts flavanones to dihydroflavonols; UFGT, the key gene in anthocyanin biosynthesis and MYBA1, its main transcriptional regulator. Although the expression trend of CHS3 was similar between C and TV in both years with a contemporary peak of expression, it was significantly higher in TV than in C (Figs 5 and 6). In 2015, F3H1 expression pattern was shared between TV and C (Fig. 5) but a greater peak was detected in TV than C during veraison (DOY 217). In 2016, the TV treatment initially delayed F3H1 activation (Fig. 6) and the expression pattern of this gene differed between C and TV, where an overexpression was detected almost ten days after C, in correspondence to the end of veraison (DOY 227). MYBA1 and UFGT expression were enhanced by TV treatment in both years, and after a peak of MYBA1 detected during veraison, a peak in UFGT transcription was successively (in 2015) or contemporarily (in 2016) detected in TV.

In TV, many genes showed different behaviour in the two years (Supplementary Figs S4 and S5) and so, even if they can contribute to anthocyanin accumulation in one specific year, their expression has to be considered the result of the interaction of low night temperature with the specific seasonal climatic condition. Among these, CHS1, F3H2, GST4 and transcripts coding for F3′5′Hs showed a statistically significant upregulation in TV only in 2016, while DFR and LDOX expression were respectively not affected and induced in 2015 but repressed by TV treatment in 2016 (Supplementary Figs S4 and S5). CHS2 was the only gene that did not show an expression modification with the treatments application in both years (Supplementary Figs S4 and S5).

Even if low night temperatures applied after veraison (TPV) sporadically modified the transcription of genes involved in flavonoid biosynthesis they were not effective in increasing the accumulation of anthocyanins. In TPV most of the genes we analysed were affected more by the season than treatment and only a few genes showed a univocal behaviour (Figs 5 and 6). Low night temperature after veraison never affected the transcription of CHS2, F3H1, F3H2, DFR, LDOX in TPV, which showed an expression level similar to C. MYBA1 and GST4 were induced in TPV only in 2015 (Fig. 5 and Supplementary Fig. S4) while in 2016 an increase in CHS1, CHS3 and F3′5′H expression and a decrease in UFGT, GST4 and MYBA1 expression were detected (Fig. 6 and Supplementary Fig. S5).

## Discussion

Considering the influence that minimum temperature has on grape metabolism^29,30,43^, and that to date most of the recent researches have focused mainly on the influence of daily temperatures on berry composition and grape coloration^39–42^, we decided to conduct a comprehensive study on cv. Corvina to assess at biochemical and molecular level the effect of night temperature on anthocyanin biosynthesis, which is essential for colour and overall quality of wines^44^.

Relations between night temperature, vine physiology and grape ripening trends were analysed, as directly related to berry metabolism and anthocyanin accumulation^45,46^. Photosynthesis is one of the main physiological processes that might be affected when vines are exposed to low temperatures^19,22^. Bertamini *et al*.^23^ reported that the most sensitive varieties display a clear photosynthetic activity reduction after exposure to cold nights. In our study, low night temperatures did not show any significant effect on photosynthesis and TSS accumulation (75 to 85% of which are sugars), regardless of the stage of application, even if in 2016 the warmer temperatures recorded in post-veraison slightly enhanced the TSS accumulation in C berries, which were exposed to ambient night conditions throughout the whole ripening.

In contrast to grape TSS accumulation, low night temperature showed a significant effect on berry titratable acidity, slowing down the acid degradation when applied around veraison. It is well known that malate is consumed by respiration during berry ripening with degradation rates increasing with temperature^47^. Respiration occurs both in the light and in darkness, so this process is sensitive to night temperatures. Interestingly, low night temperatures applied after veraison were irrelevant on acid degradation. This might be explained by the fact that respiration reaches maximum rates around veraison and decreases towards ripening^48^. Moreover, Ruffner^49^ suggested that changes in compartmentalization might be involved in the regulation of malic acid metabolism during the ripening process.

Many papers have taken into account the effect of raising daily temperature on the accumulation of anthocyanins at biochemical and molecular levels. Most of these researches, conducted not only on grapevine^40,41,50,51^, but also on other fruit species such as plum^52^, strawberry^53^ and apple^54^, agree that raising daily temperatures reduces the accumulation of anthocyanins. Final anthocyanin concentration at high daily temperature seems to depend on the counterbalance between its synthesis and degradation^41,50,52^. Instead, studies have not clearly elucidated how minimum night temperature affects the biosynthesis and accumulation of anthocyanins in grape skin. Since anthocyanin accumulation and profile in grape skin is influenced by several environmental factors, such as day temperature, illumination, water and nutrient availability and by vineyard management techniques^55–58^, in the present experiment, all factors except for minimum night temperature, were equalized among treatments and control, in order to exclude their effect on the biosynthesis of anthocyanins. This approach also allowed differences among treatments in degradation processes to be excluded, which generally start in the daytime under light and temperature over 30–35 °C^50,59,60^.

Low night temperatures were effective on anthocyanin accumulation only when imposed around veraison, while their effect after veraison was irrelevant. This is in agreement with previous studies reporting the veraison stage as being the most sensitive for anthocyanin accumulation in grape berry skins^42^. The results were confirmed in both seasons, but appeared more evident in 2015, when the veraison stage was warmer. In the latter year, difference in minimum berry temperature between treated vines and the control frequently reached up 15 °C, and changes in anthocyanin concentration between TPV and C were maintained until harvest.

Only a small effect of low night temperature was observed on the anthocyanin profile. This is an important aspect for wine quality, because individual anthocyanins have different characteristics with regard to colour or stability. Few reports analysed the effect of low night temperature on individual anthocyanin concentration in grape skin. Mori^21^ found that the ratios of delphinidin-3-glucoside, cyanidin-3-glucoside and petunidin-3-glucoside to the total anthocyanin content were greatly reduced under high night temperatures (30 °C) compared to low night temperature conditions (15 °C). The author assumed that, as methoxylation, glycosylation and acylation increase thermal stability of anthocyanins^61^, low-methylated anthocyanins, like delphinidin and cyanidin, were more sensitive to degradation than highly methylated ones (malvidin). He also suggested that anthocyanin methylation was enhanced under high night temperature conditions. Our results seem to confirm these data only in 2015 for acylated anthocyanins and in 2016 for the percentage of delphinidin 3-glucoside, revealing a weak effect of low night temperature on anthocyanin composition in Corvina cultivar.

To fully understand how anthocyanin accumulation responds to low night temperature in different berry ripening phases, the expression of genes involved in anthocyanin biosynthesis was analysed. In our research, only cool nights around veraison were effective, while the post-veraison treatment was irrelevant in increasing anthocyanin accumulation. These results were supported at molecular level in both study-seasons, despite the climatic conditions of 2015 and 2016 being very different. The genes induced by cooler nights at veraison are key genes in anthocyanin biosynthesis and participate in early (CHS3, F3H1) and late steps (MYBA1, UFGT) of anthocyanin biosynthesis. All these genes are physiologically induced in grapevine during veraison and an increase of their transcription could enhance anthocyanin accumulation^56^. Differently from MYBA1 and UFGT, whose expression is induced by light^62^, CHS3 and F3H1 expression is more related to temperature than light as their expression may be induced by lower temperatures in both dark and light conditions^42,63^. In our research, their expression increase could contribute to the rise of uncoloured substrate for UFGT for the late steps of anthocyanins production in comparison to C. Surprisingly, changes in MYBA1 and UFGT transcript levels were not temporally correlated with an increase of anthocyanin concentration, suggesting that for the entire duration of TV treatment anthocyanin synthesis was not favoured by cooler nights and we may assume that some mechanisms downstream of the increase in UFGT expression, such as UFGT enzymatic activity and anthocyanin transport inhibition, occurred and prevented the accumulation of anthocyanins. Post-transcriptional or post-translational regulation of UFGT has been proposed by several authors in the last years. In a recent research conducted on mulberry leaves^64^ a correlation analysis was conducted among the expression level of UFGT, UFGT activity and flavonoid glycosides accumulation and it was shown that UFGT activity is much more correlated than UFGT expression with flavonoid glucosides accumulation. A similar behaviour was also recorded in grapevine under high^41,50^ or low night^21^ temperatures and we can hypothesise that in our study the activity of UFGT for the entire duration of TV treatment could be inhibited by low night temperature (around 10 °C) in comparison with ambient night temperature (15–20 °C). Another aspect that could be involved in temporary lack of accumulation of anthocyanins in TV, is the decrease in their transport into the vacuole. VviGST4 belongs to the GST family, it is almost exclusively expressed in berry skins from the veraison stage and it has been described as essential for anthocyanin accumulation^65^. In both years the expression of VviGST4 in TV was lower than in C until TV was inside the climatic chamber and so it may have contributed to the reduced anthocyanin accumulation observed in TV. Once the vines were taken back to ambient night temperatures, the re-establishment of optimal conditions for UFGT activation, together with the surplus of substrates for UFGT produced by CHS3 and F3H1 during the treatment could be responsible for the detected increase in anthocyanin accumulation.

Our results suggest that the increase in expression previously reported for CHS3, F3H1, UFGT and MYBA1 genes, is directly related to low night temperatures, regardless of the daily ones. On the other hand, we can hypothesize that genes that showed a different trend of expression between TV and C in the two years were not enhanced by low night temperatures. Among these, DFR and LDOX, which act immediately upstream of UFGT, displayed a very interesting behaviour around veraison, showing a strong up-regulation in C in 2016 and opposite behaviour in 2015 in comparison to TV. Since from 7:00 to 19:00 all the vines were subjected to the same temperatures, we can assume that DFR and LDOX expression may have been favoured by the ambient air night conditions of 2016, with temperatures ranging between 15 and 20 °C, while lower or higher temperatures, as those recorded in TV or in 2015, did not exert a similar positive effect.

Following the application of low night temperature in post-veraison phase, no differences in anthocyanin accumulation were detected between TPV and C in either season. None of the four genes responsible for the increased anthocyanin accumulation detected in TV, was positively affected in TPV. CHS3 and F3H1 always displayed the same expression profile as C, while UFGT and MYBA1 showed different trends in the two seasons, with a contemporary decrease in expression in 2016. A study conducted by Yamane *et al*.^42^ showed that veraison is the most sensitive stage for anthocyanin accumulation in the berry skins of Aki Queen and that low average temperature treatment (20 vs 30 °C) during veraison significantly enhanced anthocyanin accumulation via an increase in mRNA levels of anthocyanin biosynthetic genes. Furthermore, exogenous treatments with abscisic acid^66^ or agronomic practices, such as the application of different deficit irrigation regimes^67^, removal of apical leaves^68,69^ or shoot trimming^70^ don’t imply a variation in skin anthocyanin accumulation if conducted in post-veraison. According to our results, it appears clear that the possibility of influencing anthocyanin biosynthesis at transcriptional level by low night temperature is limited to the veraison stage and that subsequently no positive effects may arise.

To the best of our knowledge this is the first comprehensive study that attempted to clarify the effect of low night temperature on anthocyanin accumulation and on the expression of genes related to anthocyanin biosynthesis. Overall, our data indicate that low night temperature (10 °C in comparison to control temperature over 15 °C) around veraison hastened berry coloration, with no effect on berry TSS accumulation or titratable acidity content at harvest. Low night temperatures during the post-veraison phase did not affect the anthocyanin gene expression or accumulation, nor berry ripening trends.

These findings lead to interesting considerations in relation to the global warming scenario, as changes in environmental conditions will necessitate adjustments to preserve the typical characteristics of wines produced in specific areas. Along with choice of plant material and agronomic management, the potential for shifts in present viticulture zones, must be considered. For many regions, the trends in growing season average temperatures are moving them from what would be considered too cool for grape growth to within the range of cool climate viticulture or even into intermediate climate suitability^71^. In our specific study-case, Corvina cultivation has traditionally been restricted below 500 m a.s.l., while the upper strip of the Verona pre-Alpine hills was considered too cold for quality grape production. Nowadays, climate trends show that sites at higher elevation, up to 800–900 m a.s.l., are displaying temperatures able to satisfy vine requirements (http://www.arpa.veneto.it), with potentials for optimum grape ripening and enhanced grape coloration. Considering anthocyanin biosynthesis, benefits may arise both by the effect of moderate temperatures and by the increased UVB radiation that characterizes sites at higher altitudes, as demonstrated by previous studies^72,73^. Therefore, researching future climates suitable for viticulture among the present-day locations and exploring vine response to these conditions may be a promising strategy for ensuring wine quality in a climatically changing future. In addition, further investigations including metabolomic approaches will surely improve the global comprehension of the mechanisms underlying low night temperature effects on grape ripening.

## Materials and Methods

### Plant material and temperature treatments

The trial was conducted in 2015–2016 at Susegana (45°84′N, 12°25′E), Italy, on three-year-old *Vitis vinifera* L. cv Corvina vines grafted to Kober 5BB rootstock and grown outdoors in 45 L pots containing a mixture of soil and peat (2:1). Eighteen uniform vines were selected from a group of thirty and during winter pruning were adjusted to one single vertically positioned cane 9–10 nodes in length. Three weeks after budburst (BBCH stage 15^74^), shoot and cluster number were adjusted to 10–12 and 8–10 per vine respectively, and six vines were assigned to each of the following treatments within a completely randomised design: low night temperature (10 °C) in veraison (TV); low night temperature (10 °C) in post-veraison (TPV); control, ambient night air-temperature (C).

TV treatment began before veraison, prior the onset of coloration and continued until full veraison, when all berries were coloured. TPV treatment started after full veraison (as formerly described) and continued for about 4 weeks. The temperature treatments were conducted as follows: potted plants were transferred into a cold room in the evening for overnight exposure (19:00 to 7:00) in darkness. Simultaneously, to standardize the photoperiod, untreated plants were placed under a shade cloth structure closed on all sides and opened in the basal segment to allow air flow. On the following morning all vines were transferred to the open air for the day according to the previously described completely randomised design to minimize different cluster sunlight exposure. The shoots of each vine were vertically positioned during elongation and no shoot trimming was performed throughout the season. Vines were well watered throughout the vegetative seasons by an automatic drip irrigation system. In order to prevent water stress conditions, daily water consumption was monitored by weighing two reference potted vines, and irrigation volumes were adjusted accordingly. Standard horticultural practices were applied for fertilization and disease control.

### Temperature measurements

Berry temperature was monitored on 3 clusters from each treatment using a WatchDog 1400 data logger (Spectrum Technologies, Bridgend, UK) equipped with three T-type thermocouples (RS Components, Milan, Italy), which were manually inserted into the berry flesh at approximately the equator of the sphere. The insertion point was sealed by silicone to prevent berry drying, and thermocouples were moved weekly to fresh berries. Berry temperature was recorded every 30 min from the start of the experiment to its conclusion. Air temperature was recorded every 30 min throughout the growing period, using an automatic weather station (Tinytag TGP 0073, Gemini, UK) positioned at the experimental site.

### Gas exchange and stem water potential measurements

Leaf net assimilation rate (An, *μ*mol CO_2_ m^−2^ s^−1^) was measured on 6 fully expanded leaves per treatment at two-time points in the two study-seasons: pre-veraison, prior the start of the experiment (DOY 198 and 204 in 2015 and 2016, respectively) and at mid maturation, concurrently with TPV treatment (DOY 244 and 249 in 2015 and 2016, respectively). One primary leaf per vine, inserted at node 4–6 above the distal bunch on a main median shoot, was sampled at each measurement. The assimilation rate was measured in the morning (09:00 to 11:00) on cloudless days using a portable photosynthesis system CIRAS 2 (PP-Systems Europe, Scandicci, IT). Measurements were performed under saturating light conditions (1600 µmol photons m^−2^ s^−1^), at a CO_2_ concentration of 400 µmol mol^−1^, and at ambient temperature. Mean leaf temperature ranged between 26.4 and 29.0 °C for all treatments at all time points.

On the same dates, midday stem water potential (stemΨ) was measured by pressure chamber following the procedure described in Choné *et al*.^75^. Measurements were taken at sun zenith on six primary leaves per treatment, selected as described for the assimilation rate measurements.

### Sampling procedure

In the two study-seasons berries were sampled from the start of the experiment to its conclusion at the following stages referred to control vines:

1. ≈ 1 week before veraison (DOY 210 in 2015 and 209 in 2016, beginning of TV treatment);
2. 50% veraison (DOY 217 in 2015 and 218 in 2016);
3. end of veraison (DOY 222 in 2015 and 227 in 2016, beginning of TPV treatment);
4. 17–19 days after full veraison (DOY 239 in 2015 and 246 in 2016);
5. 38 days after full veraison (DOY 260, only in 2015);
6. fruit maturity (DOY 267 in 2015 and 272 in 2016, defined as berry soluble solids ≥17° Brix in control vines).

Soluble solids, titratable acidity and biochemical analyses were performed in all stages, while gene expression analyses in stages 1, 2, 3 and 4. At each time point three independent pools (60 berries each) were created by randomly collecting 30 berries from each of the six vines per treatment. In total 180 berries were sampled for each treatment and date. The samples of each replicate were divided into three parts: 10 berries were weighed and used for the evaluation of soluble solids and titratable acidity. The others were immediately frozen in liquid nitrogen and stored at −80 °C for biochemical analysis (30 berries), and mRNA analysis (20 berries).

### Soluble solids, titratable acidity and anthocyanin analysis

Berries were crushed and the must sieved for soluble solids and tritatable acidity analysis. Soluble solids were measured by refractometer (Atago PR32) at 20 °C. Titratable acidity (expressed as g L^−1^ of tartaric acid equivalents) was determined using a Micro TT 2022 automatic titrator (Crison, Barcelona, Spain) by titration with 0.1N NaOH. Anthocyanins were extracted from the skin of 30 berries and analysed^76^. For each replicate, an aliquot of 0.1000 g (±0.0005 g) of frozen ground berry skin was extracted in 1.0 mL of 50% (v/v) methanol in water for 20 min with sonication. The extracts were then centrifuged (10 min at 13,000 × g) and 200 µL of the supernatant transferred to HPLC auto-sampler vials. Samples were analysed on an Agilent 1220 infinity HPLC (Agilent Technologies), equipped with a diode array detector (DAD) and a C-18 SS Wakosil (150 mm × 4.6 mm, 3 µM; SGE, Ringwood, Australia). Anthocyanins were quantified at 520 nm using an external calibration curve with malvidin-3-glucoside chloride as the standard (Sigma-Aldrich).

### RNA extraction, Reverse Transcription (RT) and Real Time qPCR

Total RNA was isolated from approximately 400 mg of pulverized berry skins from three biological replicates sampled at DOY 210, 217, 222 and 239 in 2015 and DOY 209, 218, 227 and 246 in 2016 using the Spectrum™ Plant Total RNA kit (Sigma-Aldrich), following the manufacturer’s instructions. RNA quality and quantity were determined using a Nanodrop 2000 spectrophotometer (Thermo Scientific, Wilmington, DE, USA). One microgram of extracted RNA was treated with two units of DNase I (Promega, Madison, USA) and then reverse transcribed using Improm-II Reverse Transcriptase (Promega) according to the manufacturer’s instructions. In order to assess if the cDNA had been properly produced, an amplification with primers for an Ubiquitin1 coding gene^77^ was performed. Real Time qPCR was performed using the Power Sybr Green Pcr Master Mix (Thermo Fisher Scientific), to amplify a specific region of target genes: primers for CHS1, CHS2 and CHS3 (calchone synthase 1, 2 and 3), DFR (dihydroflavonol reductase), LDOX (leucoanthocyanidin dioxygenase) and UFGT (UDP glucose:flavonoid 3-O-glucosyltransferase) were retrieved from Goto-Yamamoto *et al*.^78^; primers for F3H1 and F3H2 (flavanone 3-hydroxylase 1 and 2) and MYBA1 were retrieved from Jeong *et al*.^79^; primers for GST4 (glutathione S-transferase 4) was retrieved from Conn *et al*.^80^. Primers designed on two specific and conserved regions of plant F3′H (flavonoid 3′-hydroxylase) and F3'5′H (flavonoid 3′,5′-hydroxylase) genes^81^ were used to analyse transcripts belonging to F3′H and F3′5′H families. Real Time qPCR and analyses of Real Time data were conducted as reported in Pastore *et al*.^82^, using Ubiquitin1^77^ as reference gene.

### Statistical analysis

One-way or two-way analysis of variance (ANOVA) were conducted and, in case of significance of F test, mean separation was performed by the Tukey test, using STATISTICA 8 (StatSoft Inc., Tulsa, OK, USA), at P < 0.05, 0.01 and 0.01. Three replicates per treatment were used for biochemical and gene expression analysis, while physiological data (gas exchange and stem water potential) were analysed using six replicates per treatment. Temperature data were analysed with three replicates for each treatment.

### Data availability

The datasets generated during the study are available from the corresponding author upon request.

## Electronic supplementary material

Supplementary material
