# Supplementary material for: Low night temperature at veraison enhances the accumulation of anthocyanins in Corvina grapes (Vitis Vinifera L.)
Source: Sci Rep. 2018 Jun 7;8:8719. doi: 10.1038/s41598-018-26921-4 (PMC5992194; doi:10.1038/s41598-018-26921-4)
Supplement: Supplementary file 1 — Supplementary material [file 41598_2018_26921_MOESM1_ESM.pdf]

Supplementary material

Low night temperature at veraison enhances the accumulation of anthocyanins in Corvina grapes (*Vitis Vinifera* L.)

Federica Gaiotti, Chiara Pastore, Ilaria Filippetti, Lorenzo Lovat, Nicola Belfiore, Diego Tomasi

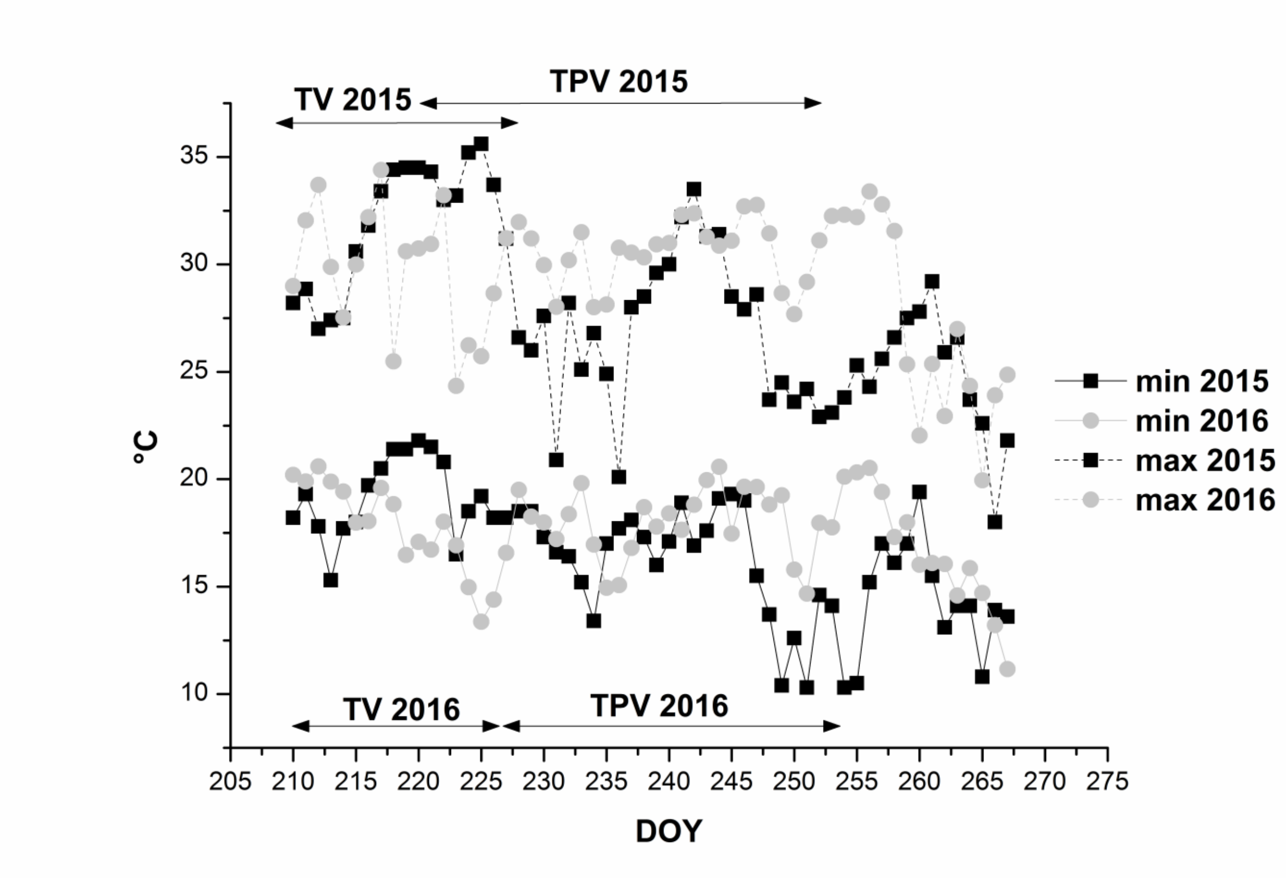

Supplementary Fig. S1. Trends in minimum and maximum air temperature from veraison to harvest in 2015 and 2016 seasons. The duration of the low night temperature during veraison (TV) or post veraison (TPV) periods is indicated by black double arrows.

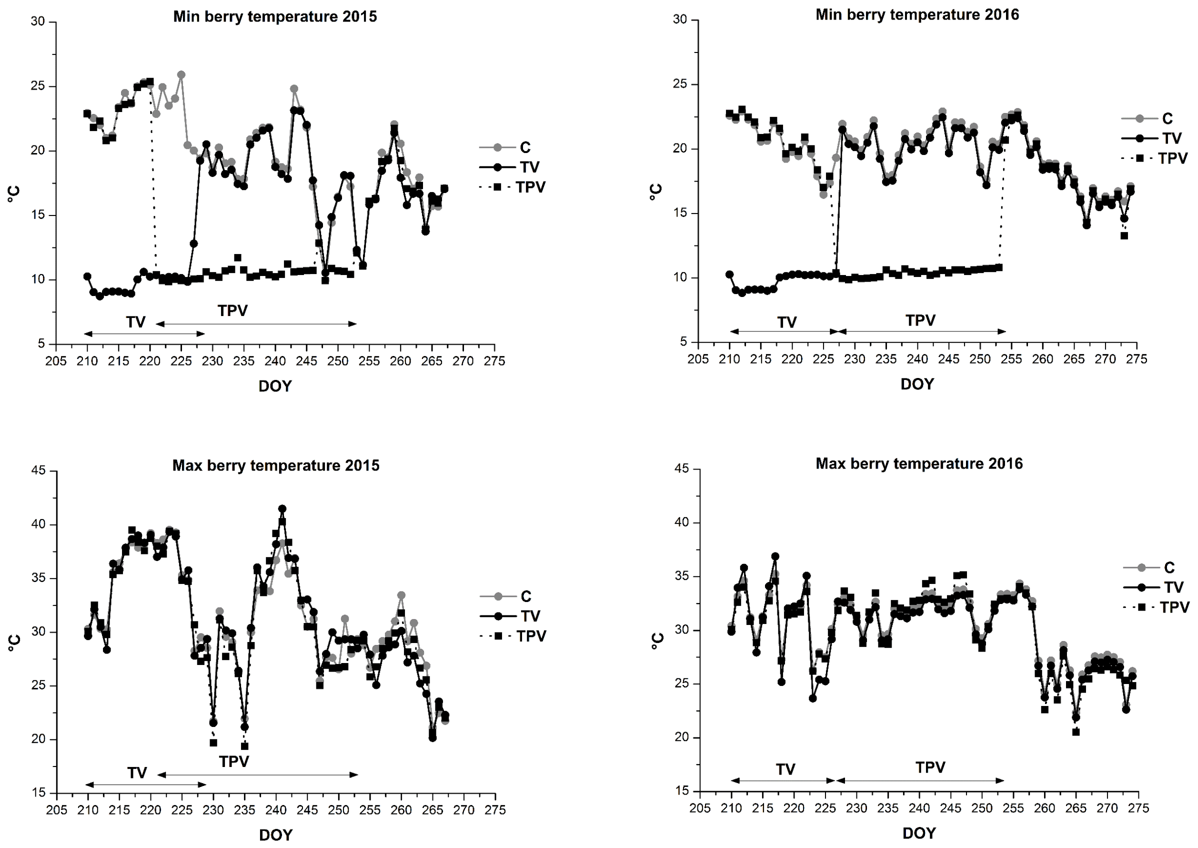

Supplementary Fig. S2. Trends in minimum and maximum berry temperature for C, TV and TPV from veraison to harvest in 2015 and 2016 seasons. The duration of the low night temperature during veraison (TV) or post veraison (TPV) periods is indicated by black double arrows.

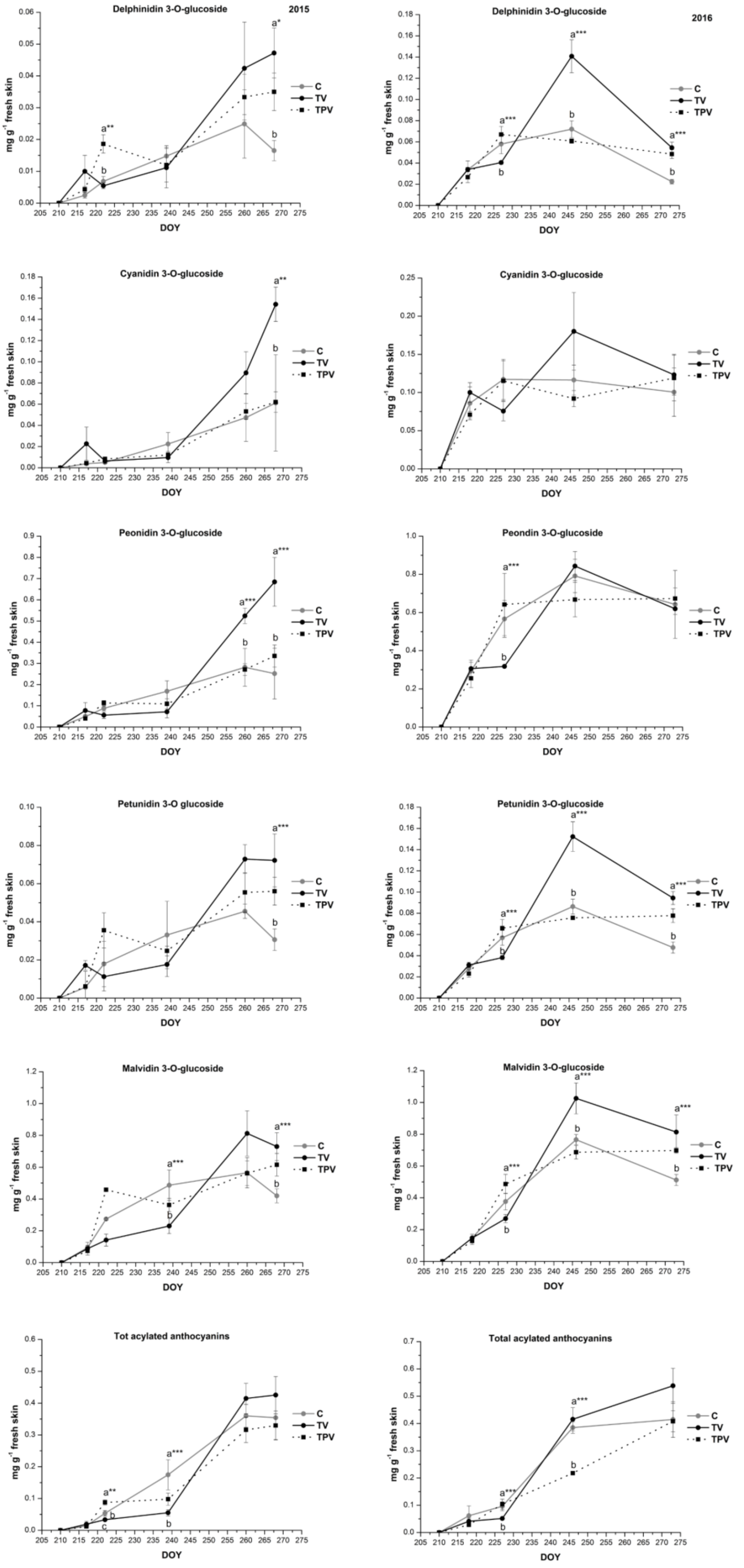

Supplementary Fig. S3. Trends of single anthocyanin concentrations in the skin of Corvina grapes treated under low night temperature (TV, TPV) and in the control (C), during the experimental period in 2015 and 2016. Error bars indicate the mean SE (*n*=3). Means followed by different letters differ significantly, as calculated by Tukey statistical analysis (*, **, *** = p ≤ 0.05, 0.01, 0.001 respectively).

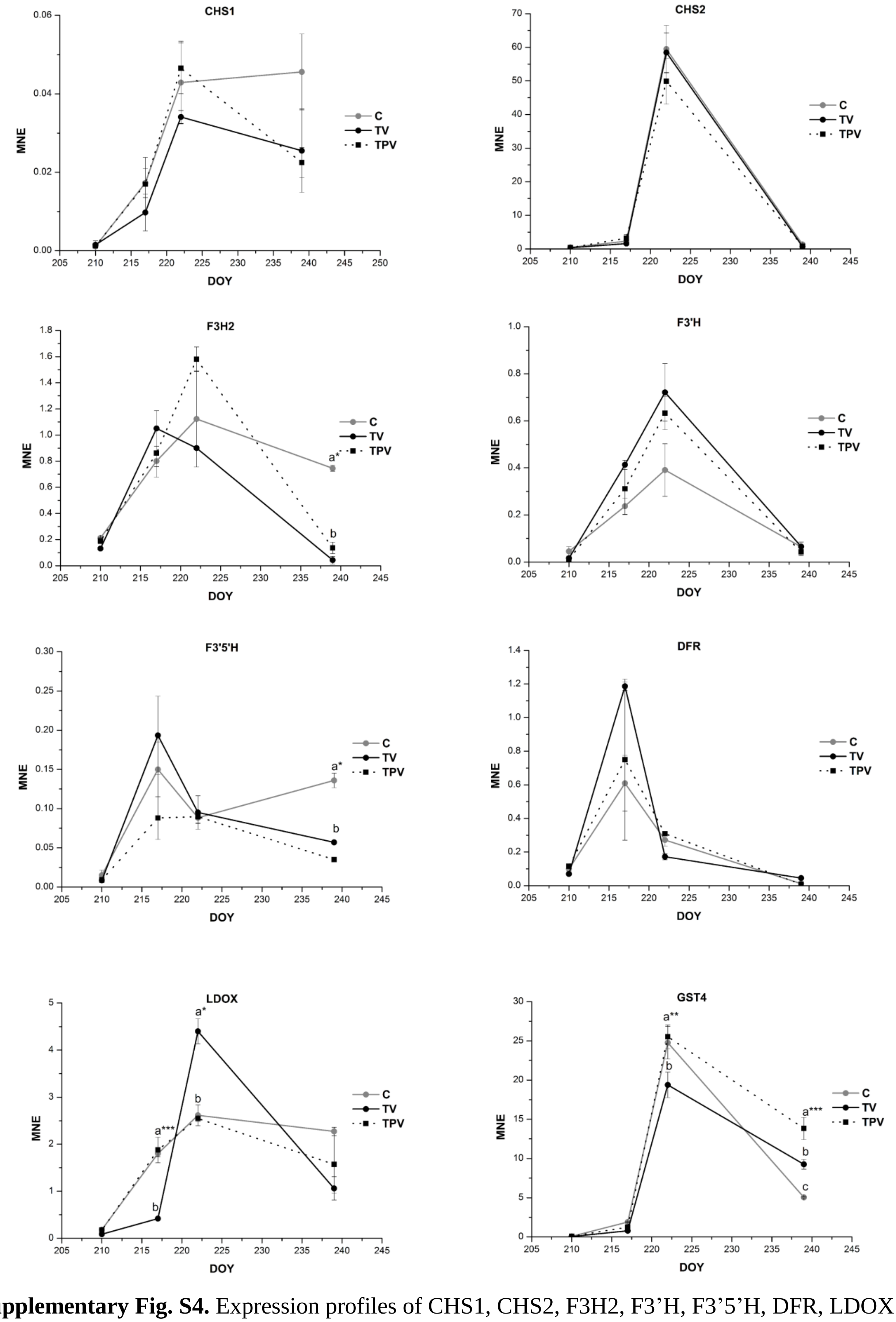

Supplementary Fig. S4. Expression profiles of CHS1, CHS2, F3H2, F3'H, F3'5'H, DFR, LDOX and GST4 genes in the skin of Corvina grapes treated under low night temperature (TV, TPV) and in the control (C), during the experimental period in 2015. Real time RT-PCR data are reported as mean normalized expression (MNE) values, obtained using Ubiquitin-1 as reference gene. Error bars indicate the mean SE (n=3). Means followed by different letters differ significantly, as calculated by Tukey test (*, **, *** = p≤0.05, 0.01, 0.001 respectively).

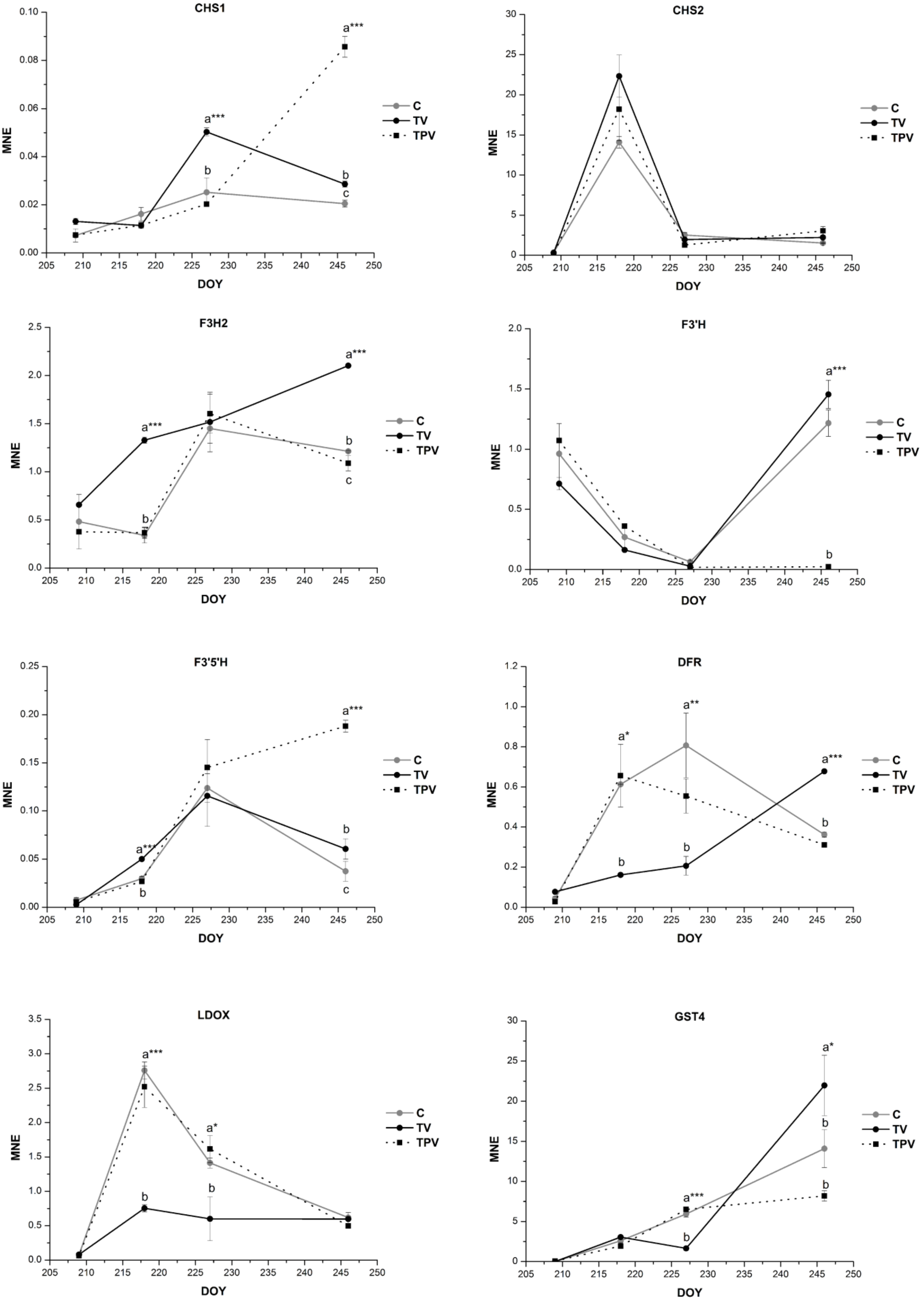

Supplementary Fig. S5. Expression profiles of CHS1, CHS2, F3H2, F3’H, F3’5’H, DFR, LDOX and GST4 genes in the skin of Corvina grapes treated under low night temperature (TV, TPV) and in the control (C), during the experimental period in 2016. Real time RT-PCR data are reported as mean normalized expression (MNE) values, obtained using Ubiquitin-1 as reference gene. Error bars indicate the mean SE (*n*=3). Means followed by different letters differ significantly, as calculated by Tukey test (*, **, *** = $p \le 0.05$, 0.01, 0.001 respectively).
